# Morphological and molecular characterization of *Calicophoron raja* (Näsmark, 1937) collected from wild Bovidae in South Africa

**DOI:** 10.1016/j.ijppaw.2022.08.003

**Published:** 2022-08-10

**Authors:** Aoi Ikeuchi, Daisuke Kondoh, Ali Halajian, Madoka Ichikawa-Seki

**Affiliations:** a Laboratory of Veterinary Parasitology, Faculty of Agriculture, Iwate University, 3-18-8 Ueda, Morioka, 020-8550, Japan; b Laboratory of Veterinary Anatomy, Department of Veterinary Medicine, Obihiro University of Agriculture and Veterinary Medicine, 2-11 Inada-cho Nishi, Obihiro, 080-8555, Hokkaido, Japan; c Research Administration and Development, University of Limpopo, P.O. Box X1106, Sovenga, 0727, South Africa

**Keywords:** Paramphistome, *Calicophoron raja*, Internal transcribed spacer 2, Molecular marker, Morphological characterization

## Abstract

Paramphistomes, commonly known as rumen flukes, are digenean parasites that infect ruminants. Accurate morphological identification of paramphistome species is challenging and often neglected. For instance, it requires sagittal midline sections of adult flukes, which are difficult to prepare. Therefore, the majority of the genetic information on paramphistomes found in the International Nucleotide Sequence Database is not supported by morphological descriptions, and the DNA barcodes of paramphistome species remain unreliable. In the present study, both morphological and molecular characterizations were simultaneously performed to ensure the reliability of the DNA information for the paramphistome species *Calichophoron raja* (Näsmark, 1937). The morphological characteristics of the sagittal and horizontal sections of adult flukes from a black wildebeest (*Connochaetes gnou*) and a waterbuck (*Kobus ellipsiprymnus*) in South Africa were identical to those previously described for *Ca. raja.* Additionally, this study represents a new host record of the species from *Co. gnou*. All sequences of the internal transcribed spacer 2 region of ribosomal DNA were 100% identical among the 18 flukes analyzed in the present study. A single nucleotide mutation was observed between *Ca. raja* in this study and *Ca. raja* detected in domestic ruminants in Kenya.

## Highlights

• The morphological species identification of paramphistomes is difficult.
• The current DNA barcoding of parapmhistomes is not fully reliable.
• Morphological and molecular characterizations should be simultaneously performed.
• *Calicophoron raja* obtained from two wild Bovidae were accurately identified.
• A new host record is provided and the species DNA barcode was deposited in GenBank.

## Graphical abstract

**Figure:**
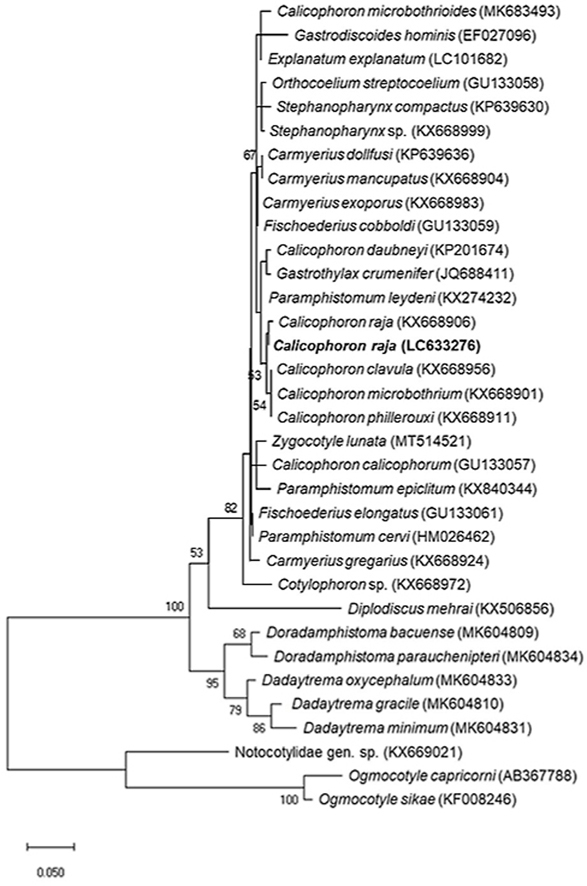

## 1 Introduction

More than 150 paramphistome species exist in mammalian hosts (Kayane, 1979). Various species of different families, especially members of Paramphistomidae, cause paramphistomiasis in ruminants (Sanabria and Romero, 2008). Most adult flukes inhabit the rumen and/or the reticulum. Immature flukes, which can cause serious morbidity and death, are found in the upper small intestine. When adult *Calicophoron* flukes parasitize the stomach, the surface of the gastric mucosa is damaged by the large acetabulum. The acetabulum encloses a plug of mucosa, the surface of which undergoes degenerative changes leading to digestive dysfunction and mucosal disease (Kayane, 1979). Both adult and immature flukes cause weight loss, and/or decreased milk production, which can lead to economic losses (Rangel-Ruiz et al., 2003).

Paramphistomiasis is diagnosed based on fecal examination and parasite egg search. However, it is impossible to differentiate between parasite species based on the morphological characteristics of their eggs alone (Sey, 1991). Therefore, morphological discrimination of adult flukes is the dominant method for species identification. However, adults have thick robust bodies, and their internal organs are difficult to visualize (Jones et al., 2005; Lotfy et al., 2010). Thus, the specific diagnosis of paramphistomes requires not only gross morphological (body shape and size as well as internal organs) but also histomorphological (structure of the muscular organs) characterization. Sample preparation is particularly important for diagnosis. Sagittal midline sections including the pharynx, terminal genitalium, and acetabulum, are required for species determination (Eduardo, 1982; Fukui, 1929; Ichikawa et al., 2013; Näsmark, 1937; Sey, 1991).

DNA barcodes of paramphistomes remain insufficient because of the difficulties associated with species discrimination in adult flukes. Most recent studies have provided molecular results without morphological descriptions. Nonetheless, this kind of molecular information alone is unreliable in the majority of cases. Therefore, morphological and molecular characterizations must be performed simultaneously (Ichikawa et al., 2013) to develop robust and reliable DNA barcoding to discriminate paramphistome species.

The internal transcribed spacer 2 (ITS2) region of nuclear ribosomal DNA is a useful genetic marker for differentiating paramphistome species (Lotfy et al., 2010; Rinaldi et al., 2005). Polymerase chain reaction (PCR)-restriction fragment length polymorphism based on nucleotide sequence variations in ITS2 has been previously used to distinguish the three paramphistome species endemic in Japan using DNA extracted from a single egg (Itagaki et al., 2003). Therefore, collecting ITS2 information as a DNA barcode for a greater number of species worldwide will enable the establishment of reliable molecular techniques to identify paramphistome species without morphological characterization.

In the present study, both morphological and molecular characterizations were performed on adult flukes of the genus *Calicophoron* collected from the rumen of two wild ruminant species in South Africa. As wild ruminants are a reservoir of paramphistomes, the DNA information provided in the present study will be a useful marker for epidemiological surveys in endemic areas.

## 2 Materials and methods

### 2.1 Adult flukes

Fourteen adult paramphistomes were collected from the stomach of a female adult black wildebeest (*Connochaetes gnou*) that had a broken leg and died at SA Lombard Nature Reserve, Bloemhof, North West province, South Africa on May 24th, 2014. In addition, two adult paramphistomes were found from the stomach of a male adult waterbuck (*Kobus ellipsiprymnus*) hunted at Polokwane Game Reserve, Limpopo Province, South Africa on Jun 19th, 2013. The paramphistomes were then relaxed and cleaned in saline, fixed, and preserved in 70% ethanol until further use. Part of the acetabulum of all flukes was sampled to determine the sequence of the nuclear ITS2 region. A portion of the left side of the acetabulum (approximately 10 mg) was removed using ophthalmic scissors and used for DNA extraction. This region does not contain DNA from other individuals, which may be found in the uterus.

### 2.2 Histomorphological diagnosis

The remaining bodies of five flukes from the black wildebeest were processed for histomorphological diagnosis. Four flukes were embedded in paraffin using standard procedures and sagittally sliced into 5 or 10 μm sections, including Laurer's canal, excretory duct, pharynx, terminal genitalium, and vitelline gland. The remaining one fluke was horizontally sliced into 5 μm sections to evaluate the distribution of the testes. Sections were stained with hematoxylin and eosin, and morphological identification was performed according to the existing classification keys described by Jones et al. (2005) and Sey (1991) at the genus level, and by Eduardo (1982) and Sey (1991) at the species level. The length and width of the flukes, as well as the size of the organs, were measured using optical microscopy and ImageJ (Schneider et al., 2012), and compared with previous descriptions by Eduardo (1982) and Sey (1991).

### 2.3 Genetic analysis of ITS2

Molecular characterization was conducted for all 16 flukes, including the five used in the morphological analysis described above. The High Pure PCR Template Preparation Kit (Roche, Mannheim, Germany) was used to extract total DNA, following the manufacturer's protocol. DNA was stored at −20 °C until further use. DNA fragments of the nuclear ITS2 region containing 5.8S and 28S partial ribosomal RNA genes were amplified by PCR using ITS2-F (5′-TGTGTCGATGAAGAGCGCAG-3′) and ITS2-R (5′-TGGTTAGTTTCTTTTCCTCCGC-3′) primers (Ichikawa et al., 2013). PCR was performed in a 25 μL reaction containing 2 μL of DNA template, 0.2 mM of each dNTP, 0.1 μM of each primer, 1.25 U of Go Taq DNA polymerase (Promega, Madison, WI, USA), and the manufacturer-supplied reaction buffer. The thermal program was as follows: 95 °C for 2 min, followed by 45 cycles of 95 °C for 30 s, 60 °C for 30 s, 72 °C for 30 s, and 72 °C for 5 min, followed by cooling at 10 °C. The PCR amplicons were purified using the NucleoSpin Gel and PCR Clean-up Kit (Macherey-Nagel, Düren, Germany) according to the manufacturer's protocol. This was followed by direct sequencing from both directions using the Big Dye Terminator v3.1 Cycle Sequencing Kit (Applied Biosystems, Foster City, CA, USA). The resultant ITS2 sequences in the present study (486 bp) were aligned with the reference sequences of paramphistomes retrieved from a previous report (Alves et al., 2020) by using the GENETYX ver. 16.0.1 software (Genetyx Co. Ltd., Tokyo, Japan). Then, outside sequences of the alignment result were trimmed to adjust the shortest reference sequences (221 bp). A maximum likelihood (ML) tree was constructed using the MEGA ver. 10.1.7 software (Kumar et al., 2018). The best fit model selected by the software to construct the tree was Kimura 2-parameter + G. Bootstrap analyses were conducted with 1000 replicates.

## 3 Results

**Fig. 1:**
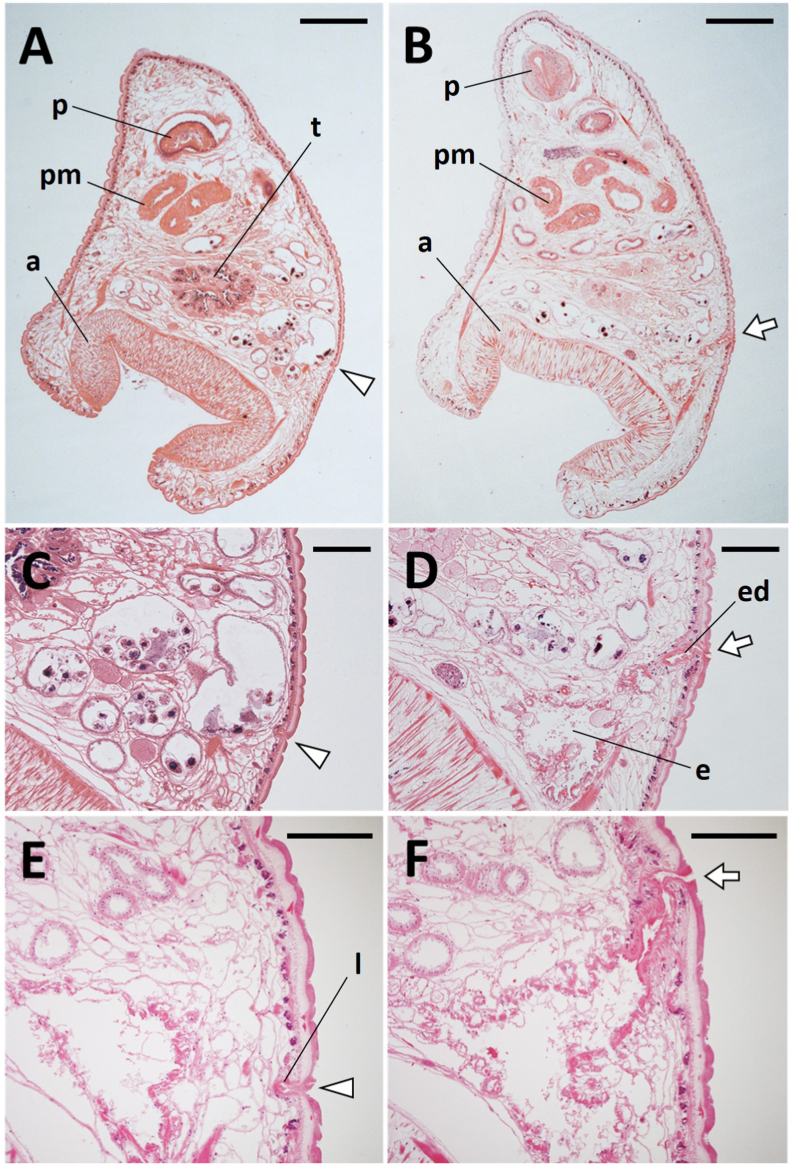
Representative sagittal sections of Laurer's canal (A, C, E) and the excretory duct (B, D, F). Arrowheads indicate the orifice of Laurer's canal. Arrows indicate the excretory pore. a: acetabulum, e: excretory bladder, ed: excretory duct, p: pharynx, pm: pars musculosa, t: testis, l: Laurer's canal. Thickness of sections: 5 μm (A, B, D-F), 10 μm (C). Scale bar: 1 mm (A, B) and 0.5 mm (C–F).

**Fig. 2:**
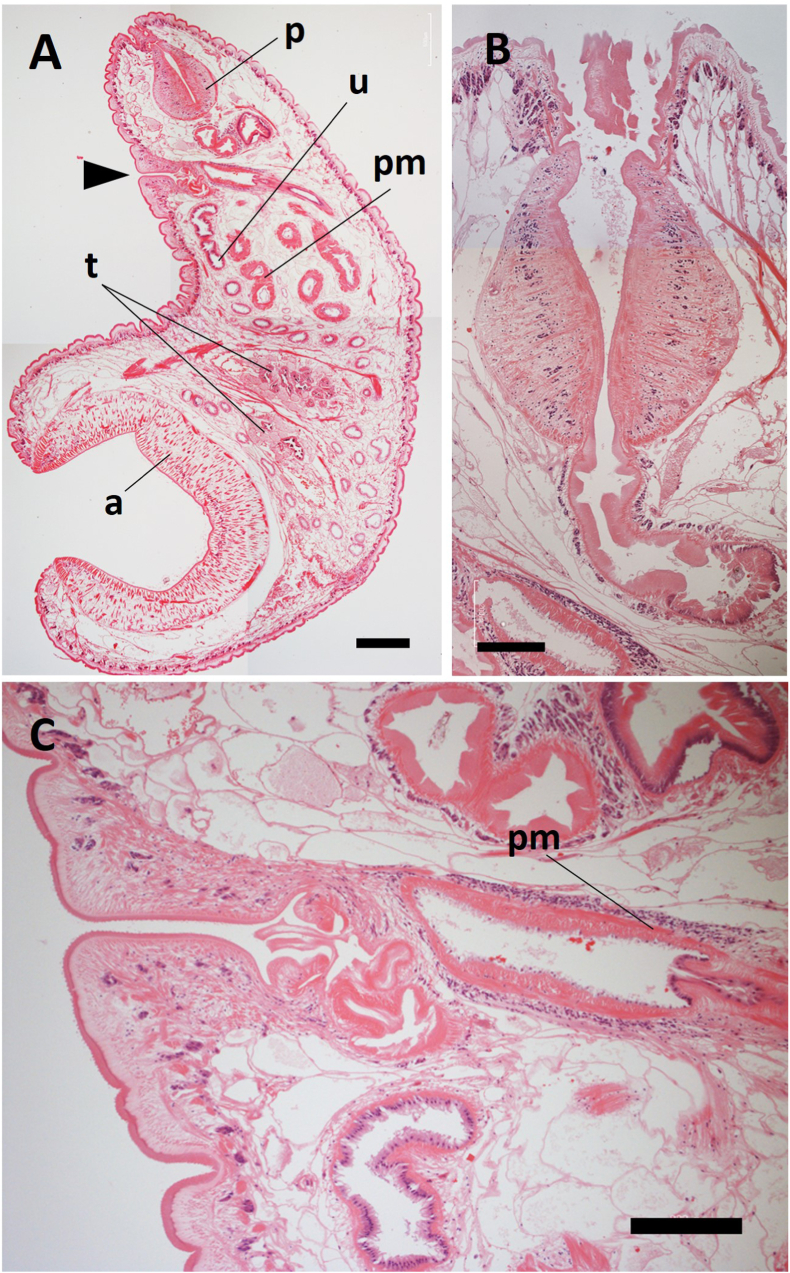
Representative sagittal sections at the level of the terminal genitalium. (A) Whole image. Arrowhead indicates the genital pore. Thickness of sections: 5 μm (A, B), 10 μm (C). (B) A pharynx of the typical Calicophoron type. (C) A terminal genitalium of the typical Raja type. a: acetabulum, p: pharynx, pm: pars musculosa, t: testis, u: uterus. Scale bar: 0.5 mm (A), 0.2 mm (B), and 0.2 mm (C).

**Fig. 3:**
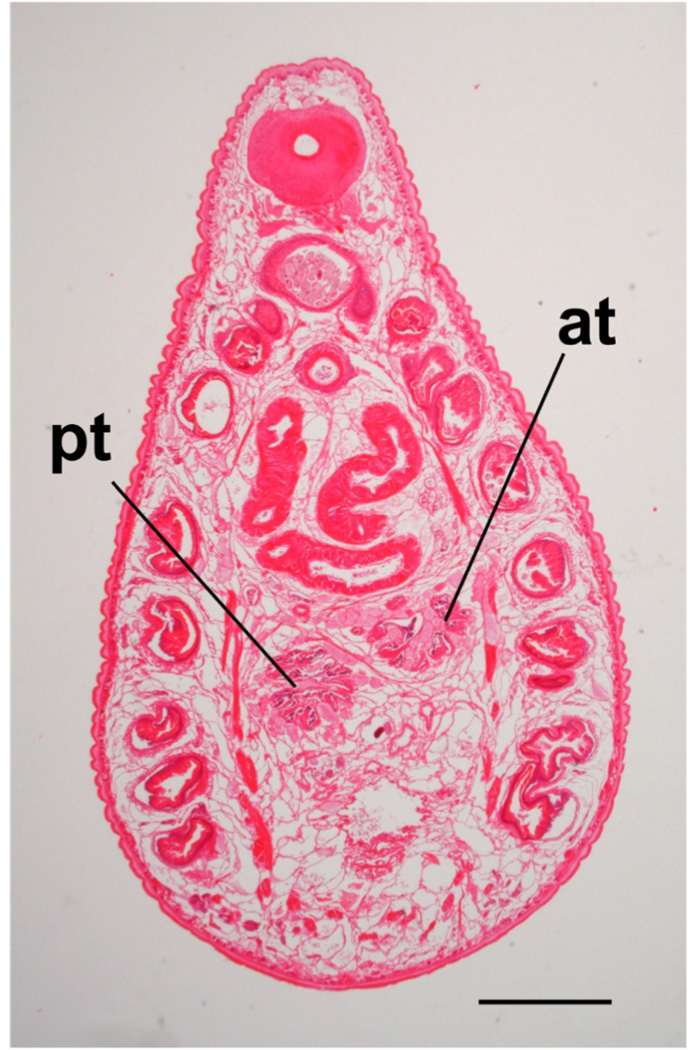
Representative horizontal section of anterior (at) and posterior (pt) testes. Scale bar: 1 mm.

The paramphistomes collected in this study belonged to Paramphistominae since Laurer's canal and the excretory duct crossed in three of the four flukes prepared for sagittal midline sections (Fig. 1). However, an orifice of Laurer's duct could not be found in the remaining one fluke, most likely due to an angle error during sectioning. The acetabulum was moderate in size, and the pars musculosa was well developed; these morphological features were matched with the genus *Calicophoron* according to Jones' (2005) classification. In addition, the pars musculosa was strongly developed (Fig. 2C) and the pharynx was of the Calicophoron type (Fig. 2B). These morphological characteristics are consistent with the descriptions of the genus *Calicophoron* in Sey's (1991) classification. The testes were not horizontally positioned in the 5 μm horizontal section of an adult fluke (Fig. 3). Furthermore, the true ventral atrium was absent, and the shape of the terminal genitalium was of the Raja type (Fig. 2C). Therefore, the *Calicophoron* flukes obtained in this study were morphologically identified as *C. raja* (Näsmark, 1937) according to Eduardo's (1982) and Sey's (1991) classifications.

Measurements of the organs using midline sagittal sections (n = 4; Table 1) were generally consistent with those described by Sey (1991). However, the size of the testes and ovaries partially deviated from the ranges described by Eduardo (1982) and Sey (1991).

**Table 1:** Morphometric values of the four *Calicophoron raja* evaluated in the present study.

	this study (n = 4^a^)	Sey (1991)	Eduardo (1982)
Body length	6.30–8.20 mm	4.10–11.20 mm	4.72–12.10 mm
Body width	3.06–4.50 mm	3.60–5.20 mm	3.39–5.05 mm
Acetabulum	2.47–3.10 mm	0.98–3.16 mm	1.11–3.36 mm
Pharynx	0.90–1.07 mm	0.39–0.98 mm	0.47–1.05 mm
Esophagus	0.68–1.01 mm	0.69–0.76 mm	0.70–0.73 mm
Anterior (left) testis			
long diameter	1.10–1.31 mm	1.98–3.26 mm	2.03–3.02 mm
short diameter	0.55–0.79 mm	1.16–1.68 mm	1.29–1.74 mm
Posterior (right) testis			
long diameter	0.95–1.35 mm	2.16–3.68 mm	2.03–3.78 mm
short diameter	0.62–0.76 mm	1.28–2.13 mm	1.37–2.03 mm
Ovary			
long diameter	0.29–0.41 mm	0.38–0.79 mm	0.45–0.87 mm
short diameter	0.18–0.31 mm	0.49–0.73 mm	0.58–0.72 mm

a The four flukes found from a black wildebeest.

**Fig. 4:**
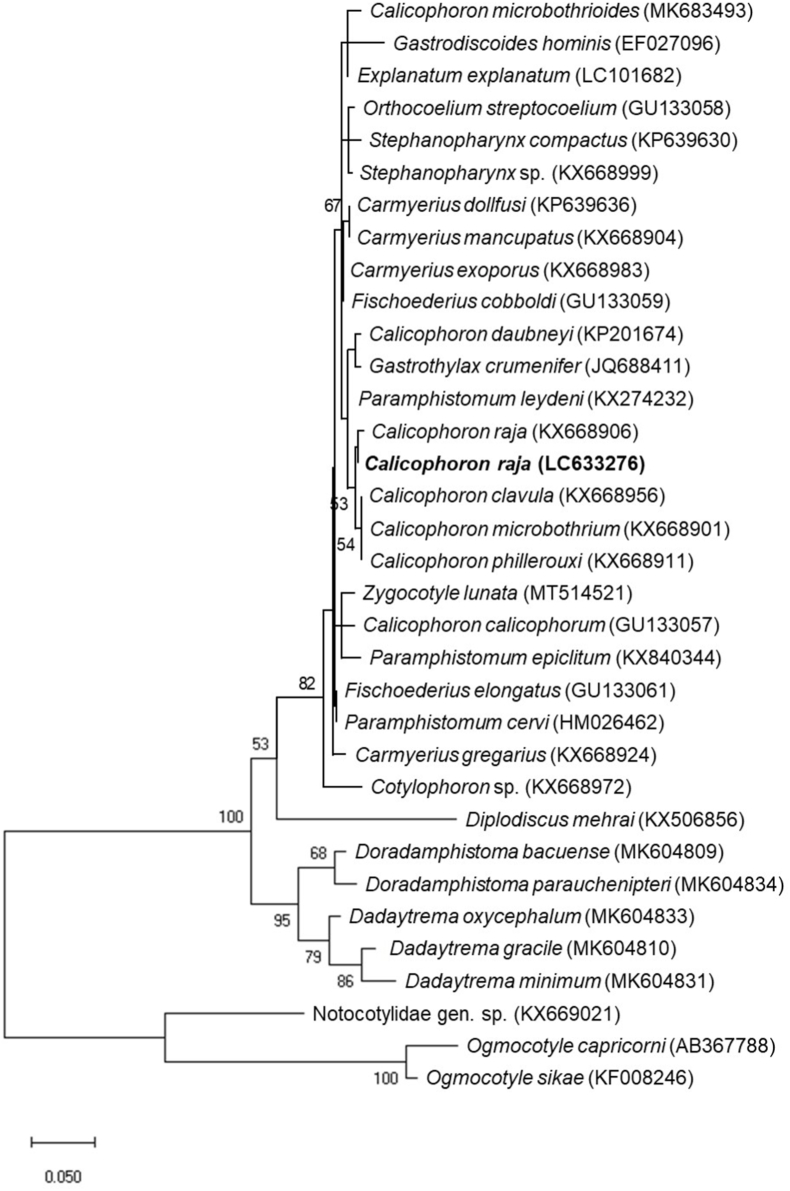
Maximum likelihood (ML) phylogenetic tree based on the internal transcribed spacer 2 (ITS2) nucleotide sequence of ribosomal DNA. Bootstrap values above 50% are displayed. *Calicophoron raja* sequences are shown in bold font. All 16 flukes obtained from a black wildebeest and a waterbuck showed an identical sequence, and the representative sequence was deposited in the INSD under accession no. LC633276.

The nucleotide sequences in the ITS2 region (486 bp) were identical among all 16 flukes analyzed in this study, and all samples collected from the black wildebeest and the waterbuck were of the same species, namely, *C. raja*. The representative sequence is registered in the International Nucleotide Sequence Database (INSD) (accession number: LC633276). In the phylogenetic ML tree, the nucleotide sequence of *C. raja* in the present study was different from the reference sequences of paramphistomes but was closely related to the members of *Calicophoron* (Fig. 4). Single nucleotide substitutions were observed between the ITS2 sequences of *C. raja* in this study and those detected in cattle, goats, and sheep in Kenya (Laidemitt et al., 2017; Pfukenyi and Mukaratirwa, 2018)*.*

## 4 Discussion

The majority of previous studies on the DNA sequences of paramphistomes did not include the preparation of sections for morphological characterization. They described only the gross morphology (shape of the flukes), and, in some studies, no morphological description was found (Jadav et al., 2018; Sanabria et al., 2011). Descriptions of gross morphology alone are not adequate when the DNA sequence is to be deposited in the INSD. In the present study, the morphological characteristics of the five paramphistomes collected from black wildebeest in South Africa were analyzed. The paramphistomes were identified as *C. raja* using accurate sagittal and horizontal sections prepared for morphological identification. However, the morphological measurements differed slightly from those described in previous reports (Eduardo, 1982; Sey, 1991). Morphometric analyses are often affected by various factors, including the developmental stage of the paramphistomes, fixation status (flaccid or contracted), and slight angular changes during the sagittal sectioning stage. Therefore, it is not possible to identify the paramphistome species based on measurements alone (Ichikawa et al., 2013).

*Calicophoron raja* has been reported in Cuba and African countries, including Kenya, Tanzania, Chad, South Africa, Botswana, Zambia, Namibia, Zimbabwe, and Sudan (Pfukenyi and Mukaratirwa, 2018; Sey, 1991). The definitive hosts reported in Africa include domestic (cattle, goats, and sheep) and wild ruminants (*Aepyceros melampus*, *Alcelaphus buselaphus*, *Connochaetus taurinus*, *Damaliscus korrigum*, *Damaliscus lunatus*, *Gazella thomsoni*, *Kobus ellipsiprymnus*, *Kobus leche*, *Kobus vardonii*, *Oryx gazella*, *Redunca redunca*, *Syncerus caffer*, *Taurotragus oryx*, *Tragelaphus strepsiceros*, *Tragelaphus scriptus*, and *Hippotragus niger*) (Pfukenyi and Mukaratirwa, 2018; Sey, 1991). This is the first study to report *C. raja* from a black wildebeest (*C. gnou*)*.*

*Calicophoron raja* infects a wide variety of species in the Bovidae. Some bovids migrate long distances annually covering approximately 3000 km in search of adequate pasture and water. This behavior may influence the prevalence and burden of parasites (Mijele et al., 2016). The results of the present study clearly demonstrate that wild animals can be reservoirs of *C. raja* in endemic areas, and domestic ruminants are at a risk of infection in mixed environments. Epidemiological studies are important to better understand the transmission dynamics of these parasites.

Single nucleotide substitutions were detected between *C. raja* from South Africa (LC633276; present study) and Kenya (KX668906). This intraspecific variation may reflect differences in host animals or geographical locations. Further epidemiological studies are required to reveal the genetic variation in this species among African regions. The transmission of the species from wild to domestic ruminants in South Africa should be further investigated since *C. raja* was found in livestock in Kenya in a previous study (Laidemitt et al., 2017; Pfukenyi and Mukaratirwa, 2018). The molecular information reported in this study could help advance future epidemiological research on *C. raja* in domestic and wild animals.

The topology of the ML tree (Fig. 4) was not clear in this study. One of the causes of this unreliability may be the short-length sequences (221 bp) used in the tree construction. However, to find reliable sequences of paramphistomes in the INSD is difficult since most of them were not supported by the morphological identifications. Therefore, simultaneous morphological and molecular characterization should be continuously performed for many paramshisomes to develop a reliable DNA database and to construct a robust phylogenetic tree of paramphistomes in the future.

## Declaration of competing interest

The authors declare that they have no known competing financial interests or personal relationships that could have appeared to influence the work reported in this paper.
